# A window into pain: American Indian cancer survivors’ drawings

**DOI:** 10.3389/fpain.2022.1031347

**Published:** 2022-10-21

**Authors:** Felicia S. Hodge, Tracy Line Itty, Rachel H.A. Arbing, Christine Samuel-Nakamura

**Affiliations:** ^1^ School of Nursing, University of California Los Angeles, Los Angeles, CA, United States; ^2^ Fielding School of Public Health, University of California Los Angeles, Los Angeles, CA, United States

**Keywords:** pain, cancer, drawings, pictures, American Indian, pain assessment

## Abstract

In order to explore the cultural constructs of pain, a series of focus groups were held among adult American Indian (AI) cancer survivors and their caregivers in the Southwest USA. Thirteen focus groups held at four sites (reservation, urban setting, hospital and clinic) elicited information on the barriers to cancer pain management. In response to facilitator questions about cancer pain and existing measurement scales, participants drew pictures to better explain their pain type (i.e., “pounding”), intensity (i.e., “spider web-like”), and other more abstract aspects of their pain episodes. Noting this novel outlet, subsequent groups were prompted for illustrations of pain. A total of 17 drawings were collected from focus group participants. We discuss seven of the drawings that best opened a window into the lived experience of pain, reflected through the eyes of cancer survivors. This study provides evidence that self-expression through color, imagery and written personal accounts provides more accurate depictions of pain for Southwest AI cancer survivors than pain scales alone. It is hypothesized that cultural modes of communication (i.e., storytelling) and intergenerational influences of artwork led to the depiction of pain in drawings. Suggestions for further exploration of the use of the pain drawings for pain assessment in healthcare settings are included.

## Introduction

Chronic pain occurs as a major symptom of cancer and cancer treatment. Although pain is prevalent among most cancer survivors, effective treatment and management of cancer pain merits more attention (1, 2). Numerous studies have reported that minority groups, including American Indians (AIs), are more likely to have under-treated pain (3) and experience or encounter patient-related, provider-related, and pharmacy-related barriers to pain management (2). Barriers to cancer pain causing fragmented management can include language obstacles, high medication costs, health care system complexities, and difficulty in obtaining care and filling prescriptions (4). The experience of pain can vary based on sex, age, spiritual and cultural beliefs, and even the context in which pain is experienced.

The Centers for Disease Control and Prevention (CDC) report that American Indians and Alaska Natives (AI/AN) are more likely to be diagnosed with certain cancers compared to non-Hispanic Whites (5). Rates of lung, colorectal, liver, stomach, and kidney cancers are reported to be higher among AIs than non-Hispanic Whites, particularly in the six regions where large numbers of AI/ANs reside (5). For instance, when AI/AN cancer rates per 100,000 people are compared against non-Hispanic Whites, liver cancer is twice as high (18.1 vs. 7.1) and cancers of the stomach (9.9 vs. 5.1) and kidney (29.8 vs. 16.5) are almost double. Reported rates per 100,000 people for AI/AN lung cancer (62.6 vs. 57.5) and colorectal cancer (50.7 vs. 36.0) also exceed rates for non-Hispanic Whites (6).

The treatment and management of cancer and cancer-related symptoms, such as cancer pain, is important for successful cancer care and quality of life. Good patient-provider communication is essential for proper diagnosis, assessment and treatment monitoring. Differing perception of pain etiology, pain experiences, and pain descriptors—particularly the context in which pain is experienced, affect successful communication between patient and provider. The experience of pain can vary widely, and the lack of good communication between patient and provider can result in limited or under-treated cancer-related pain. This paper reports on findings, specifically pain drawings, from focus groups held during the course of an intervention study on cancer pain management among Southwest AIs. The study sought to identify the types of cancer-related pain (burning, stabbing, throbbing, etc.), measures of pain, and the cultural constructs of pain. Initial focus groups gathered information on cancer pain experiences for the purposes of testing a cancer symptom management toolkit. Participants were asked to discuss their cancer-related pain experiences; however, they were initially reluctant to respond to the pain scales/figures, citing difficultly in placing numerical values to their pain. They commented that the Wong-Baker FACES® Pain Rating Scale (7) was “child-like” and appeared more “fearful” than painful. Several participants voluntarily decided that in order to best articulate the type of pain they experienced and its intensity, a drawing would be more appropriate rather than having to numerically rate or verbalize a reflection that was difficult to put into words. A group of survivors then submitted their drawings of their personal depiction of pain to the focus group facilitator for consideration in this study. The importance of imagery, as utilized in traditional native storytelling, was hypothesized to be a helpful framework for extrapolating meanings behind the drawings.

## Materials and methods

This qualitative study was part of a NIH-supported randomized control trial (RCT) that tested a cancer symptom management intervention among Southwest AI cancer survivors. To better understand participants' cancer symptom experiences (both physical and non-physical), a series of focus groups were held a year prior to the development and implementation of the intervention. The resulting intervention consisted of a Cancer Symptom Management Toolkit that included an educational film and Talking Circles educational curriculum and materials for Southwest (SW) AI cancer patients, survivors and their families. This paper reports solely on the pain drawings collected during the Focus Group phase of the study.

### Study population and recruitment

Thirteen focus groups conducted in three urban and two reservation communities recruited one hundred thirty-two (*N* = 132) adult cancer survivors, many accompanied by their caregiver. All participants were aged 18 years or older, living in a Southwest USA state, self-identified as AI, with a medically documented cancer diagnosis. The majority of participants were female (95 females and 37 males). Three focus groups were held at each of the three urban sites, and two focus groups each were held at two rural reservations located in the area. Seventeen participants (*n *= 17) from the final four focus groups drew their depiction of cancer pain on paper, which were then signed, dated and submitted to the focus group facilitator. The pain illustrations were drawn by 16 females and one male. No other additional demographic, cancer-type, or comorbidity data were collected on this subset of participants. The smaller sample size was considered adequate, considering the exploratory nature of the prompts, and enabled in-depth, case-by-case interpretation of each drawing (8).

Through a process of collaboration and agreement with rural and urban Indian health clinics, community centers, and reservation sites, adult AI cancer survivors were targeted for recruitment in the study. Recruitment lasted approximately two months and proceeded by way of word of mouth and recruitment flyers posted at community centers and clinic sites. The flyers provided information on the study, recruitment processes, and enrollment steps. Potential participants were informed of the focus group opportunity and were told if interested to sign up with the focus group moderator *via* a telephone call or in person at the clinic to reserve a space in the session. Contact individuals were accessible at the local sites and had information on the goal of the study, focus group sites and processes, recruitment and participant enrollment steps.

### Description of focus groups

Tribal approvals to conduct the study were obtained prior to the implementation of the study. Institutional Review Board (IRB) approvals were also obtained from the research organization and from the local Indian Health Service that ensures the ethical conduct of research. Following recruitment and confirmation of eligibility, individuals were informed in person and in writing of the purpose of the study, that their participation was voluntary, and that they could withdraw at any time without any negative repercussions. Further, results of the study and publications were presented to tribal leaders and publications were approved prior to planned distribution, thus supporting the concept of community/tribal ownership of the research findings and publications. Consent forms were distributed, explained, and then signed and dated by participants prior to beginning the focus group. Each focus group was composed of 12–16 members. Groups ran for approximately one/one-half hours and were facilitated by a research moderator with an assistant to monitor the tape recorder and take any necessary notes. All moderators received training in focus-group implementation in AI populations. Particular attention in training was made to the cultural concept of pain (not to place personal values on the measure or identification of pain). Strict confidentiality was maintained for the duration of this project and participants were asked to use pseudonyms to protect their privacy. Focus group sessions were audiotaped and the tapes were transcribed verbatim to ensure accuracy and systematic analysis of the discussions. Refreshments were offered to participants, as is the custom at AI gatherings. Participants received a gift card for travel and other costs associated with participating in the sessions.

Storytelling methods (9) were employed in the focus group to facilitate a better understanding of the cancer pain experience. Participants took turns telling the story of their cancer diagnosis and cancer-related pain, the types of cancer-related pain they experienced (burning, stabbing, throbbing, etc.), measures of pain they used with providers, and the cultural constructs of pain they would like to share. Responses highlighted the pathways of communication among family, friends, and communities regarding the cancer diagnosis, revealing that they “don’t talk about it.” The stories moved on to methods of pain management (pharmaceutical, heat/cold, massage, etc.), cancer etiology beliefs, treatment protocols, and methods of self-care. Participants were asked to review and comment on the study project's educational materials and information as well. Participants were asked to provide their feedback on size, font, color, cultural appropriateness, helpfulness, and any gaps that they thought should be addressed. Facilitators provided access to colored pencils in case individuals wanted to make physical suggestions for any of the materials.

In response to facilitator questions about cancer pain and existing measurement scales, some participants spontaneously drew pictures to better explain their pain type, intensity, and other more abstract aspects of pain episodes and pain management. Noting this novel outlet, subsequent groups were prompted for illustrations of pain. Facilitators directed the focus groups that written annotations could also be included. Colored pencils were used by some participants to draw their depiction of cancer pain on paper, 17 of which were signed, dated and submitted to the focus group facilitator. Fifteen of the drawings also had the participants' description of what the drawing was meant to portray written on the backside of the paper drawing. Four illustrations that were collected from participants were drawn by caregivers, and 13 pain drawings were created by female cancer survivors, with 12 of these having accompanying annotations. The integrity of all drawings and descriptions was strictly maintained; as such, no additional descriptions or clarifications were added by facilitators or researchers.

### Analysis of pain drawings

Items and groupings from the McGill Pain Questionnaire (MPQ) were used as a starting point to analyze the cancer-related pain drawings; this is a tool commonly used by the Indian Health Service as well as by healthcare providers worldwide (10). The MPQ is a self-reporting tool for pain measures among patients diagnosed with cancer and other chronic diseases. It measures both the quality and intensity of pain. Our analysis approach was to use the MPQ's four descriptors to categorize the pain drawings into four groupings:

Sensory: flickering/beating, jumping/shooting, pricking/lancinating, sharp/lacerating, pinching/crushing, tugging/wrenching, hot/searing, tingling/stinging, dull/heavy, tender/splitting
Affective: tiring/exhausting, sickening/suffocating, fearful/terrifying, punishing/killing, wretched/blinding
Evaluative: annoying/unbearable
Miscellaneous: spreading/piercing, tight/tearing, cold/freezing, nagging/torturing

Five researchers (an MD, two PhDs, and two MPH degreed researchers), two of which were AIs from the Southwest, two non-Hispanic Whites from eastern Canada, and one non-Hispanic White from the SW, ascribed each participant's depiction of their pain experience into one of the four groupings. The evaluators individually assessed each drawing using specific criteria set by the principal investigator. By continually reviewing and exploring the possible meaning of each drawing, overarching themes emerged that were mapped onto MPQ groupings (assigned with a primary, but also secondary designation, where applicable). Consensus was reached when an illustration was categorized into one or more groupings by the majority of evaluators. Although each grouping was initially mutually exclusive, two of the 13 drawings bridged pain categories so they were placed in each of the two descriptive groupings. Rigor was ensured throughout the process by using Shenton's strategies for ensuring trustworthiness (11), specifically cross-checking inferred meanings with AI researchers to demonstrate credibility, transferability, confirmability, and dependability. The process of analysis drew support from annotations by authors that were included with their drawings.

### Guiding theory

Survivors' pain depictions in drawings and the individual explanations behind these pictures were also explored using tenets of humanistic theory. The theory asserts that, “each person responds differently to matters of self, others and the environment. Indeed, each person faces the end of life in a way that represents his or her unique life experience in the world” [(12) *p*. 472]. The theory recognizes that individuals are unique and exist in the current situation, but also have the capacity to interact with others to find meaning, and that each individual has the ability and freedom to respond to the current situation through self-reflection and interpret their experiences (13). The call-and-response of the provider towards the patient allows the meeting of unmet health-related needs of patients. These needs may include relevant assessment and interventions that promote wellbeing in the face of advanced disease. Further, the theory emphasizes the concept of community and its inclusiveness of family members, colleagues, and other health care providers in the lived-experience and meeting of health-related needs (13).

## Results

Each drawing depicting a participating cancer survivor's experience with cancer-related pain was reviewed and placed into one or more of four groupings based upon MPQ categories. Table 1 shows the frequency of the 13 survivors' drawings by pain category: sensory, affective, evaluative, and miscellaneous. Table 2 displays frequency of evaluator's pain category assignment(s) for each illustration. Seven cancer survivors' drawings that depicted unique pain experiences are provided herein: three examples of sensory drawings, two examples of affective drawings, and two examples of miscellaneous drawings.

**Table 1:** Frequency of pictures of pain by category.

Category	Frequency
Sensory	9
Affective	4
Evaluative	0
Miscellaneous	2^a^

^a^ Also captured in the Affective category.

**Table 2:** Illustration assignment to pain category by rater frequency.

Picture #	Pain category:			
	Sensory	Affective	Evaluative	Miscellaneous
1	2	5	0	0
2	5	1	0	2
3	0	4	0	4
4	n/a	n/a	n/a	n/a
5	5	0	0	2
6	5	0	0	2
7	5	0	0	1
8	5	1	0	1
9	4	1	0	0
10	4	2	0	0
11	1	2	0	2
12	4	2	1	0
13	n/a	n/a	n/a	n/a
14	n/a	n/a	n/a	n/a
15	4	1	1	1
16	0	4	1	2
17	n/a	n/a	n/a	n/a

Reflects ratings from five evaluators.

**Figure 1:**
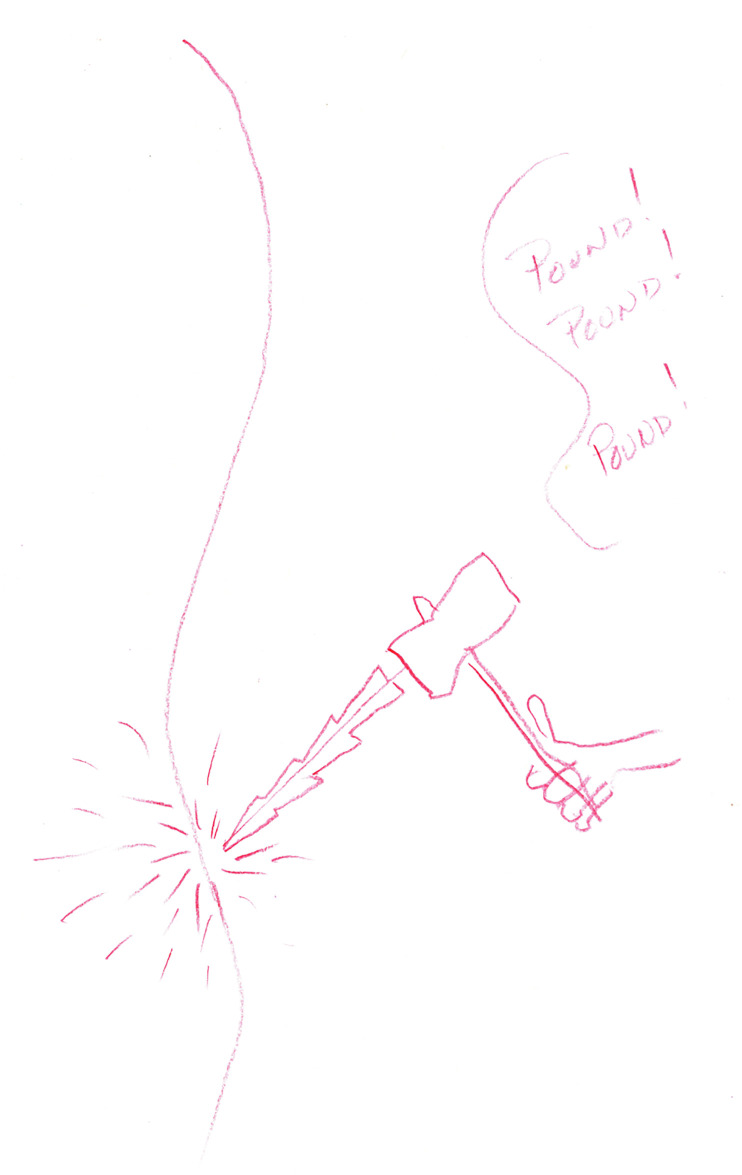
Pain as a red hammer.

The sensory category held the largest number of drawings. Nine out of 13 drawings depicted sensory pain as flickering/beating, jumping/shooting, pricking/lancinating, sharp/lacerating, pinching/crushing, tugging/wrenching, hot/searing, tingling/stinging, dull/heavy, and/or tender/splitting. An example of one of the drawings in this category is displayed in Figure 1, where drawing #5 illustrates a hammer that is pounding and reports pain that “feels like constant hammering … Pound … Pound … Pound.” The artist of this pain depiction chose to use a red colored pencil for the entire drawing to demonstrate the impact of the pain.

**Figure 2:**
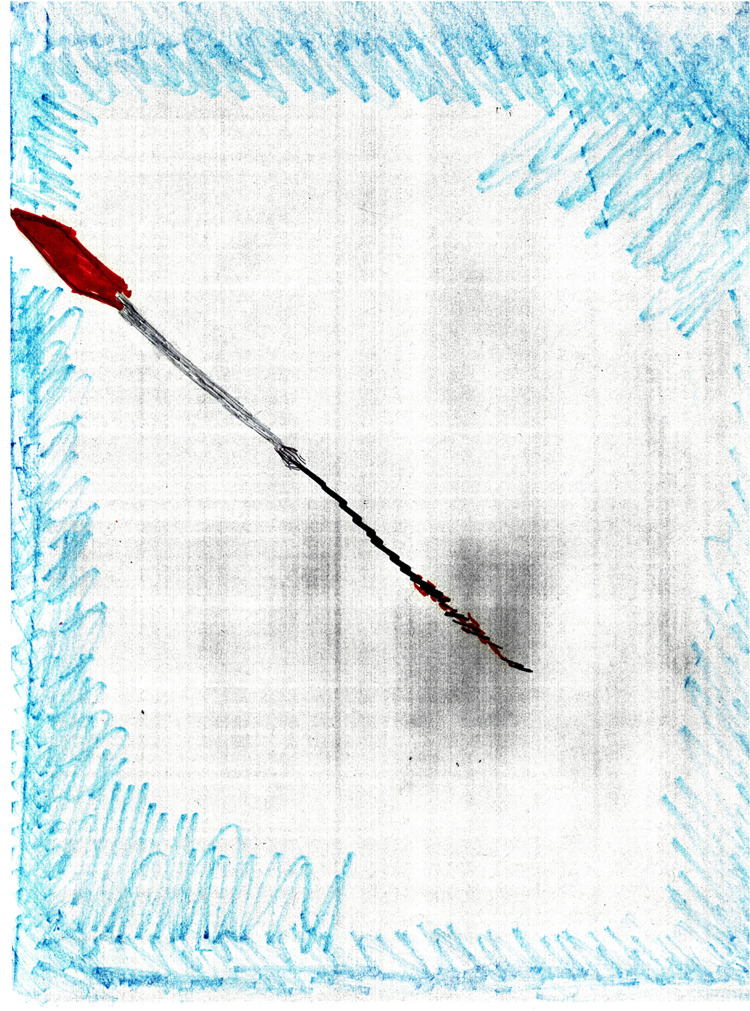
Pain as a piercing screwdriver.

Many of the drawings included use of color to demonstrate the intensity of the pain, lines, and descriptors provided in writing on the back of the paper drawing for personal measurement. Use of such sensory descriptors as “pounding” and “screwdriver-inflicted” pain is visually strong and convey well how the pain must have felt to an observer of the picture. The etiology of cancer-related pain was identified by the comment: “The cancer and the knife (referencing surgery) were the cause of my pain.” Drawing #7 (Figure 2) describes a “grayish part of my breast. The dark area in the middle of the picture is where they removed the cancer. Pain is like a screw that a Phillips screwdriver is screwing down to my inner breast. The other end is the sharp of a red knife that they removed the cancer. The red is for the slight blood during surgery.” Interestingly, the end that this survivor explains as the sharp knife is bright red. The stem of the “screwdriver” is colorless and exaggeration is depicted using darker and lighter shades of black. The slight border around the picture has uneven edges and is a faint blue color.

**Figure 3:**
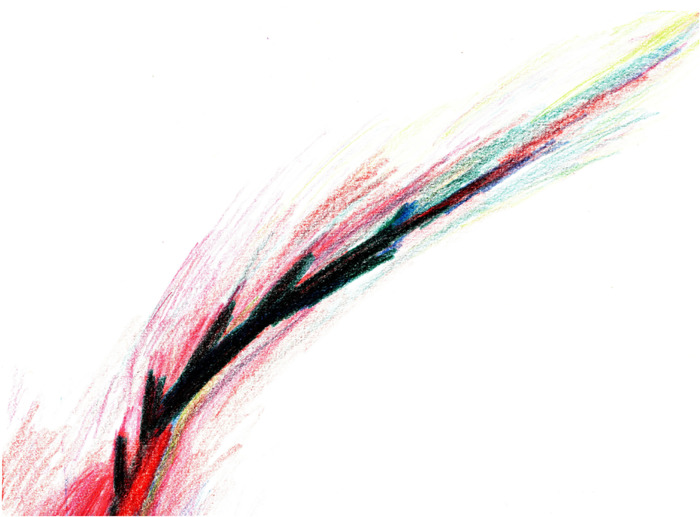
Pain as a multi-colored dark branch.

In another example of a sensory illustration, one survivor described their pain intensity (Figure 3), “The bottom is pain, but it is not a straight line. It has all these little branches along the way. The color is intense at the bottom but then it gets bigger and there is more to it at the top.” The base of the branch in this drawing is a bold red color and the rest of the branch is very dark blue and green, almost looking black. At the end of each shoot off the branch, the artist included many whisks of color, mainly red, but also including green, blue and yellow, perhaps illustrating how pain can feel differently at different times and in different places.

**Figure 4:**
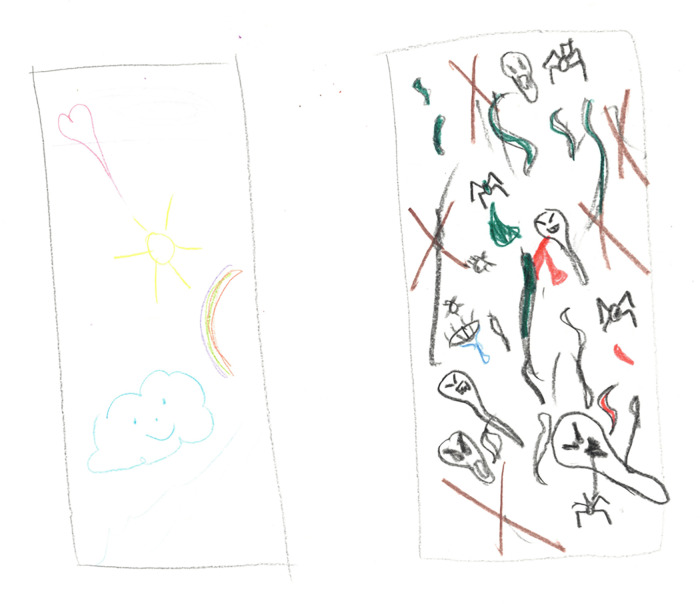
Pain as angry monsters.

The affective category was defined as pain experienced as tiring/exhausting, sickening/suffocating, fearful/terrifying, punishing/killing, or wretched/blinding. In drawing #1 (Figure 4), one cancer survivor describes the pain as “… many different types of angry monsters (that) were attacking me all over and in every direction.” The cancer survivor drew small figures moving across the page in a menacing manner.

**Figure 5:**
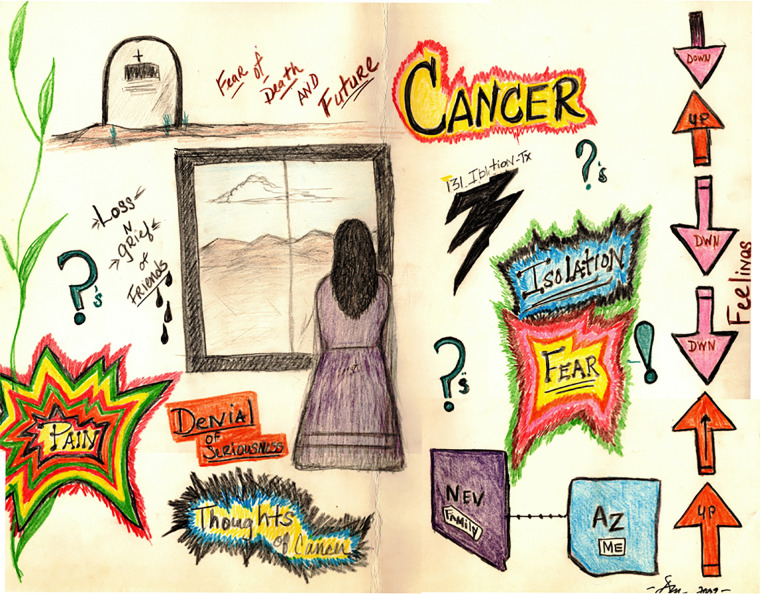
Pain caused by many factors.

An additional drawing (#16, Figure 5) presents a picture of various scenes with notations highlighting the words “Fear,” “Isolation,” “denial of surious (serious),” and “Loss N grief of Friends.” The terms “fear” and “pain” are outlined several times in bright colors such as red, green and yellow. Additionally, the survivor outlined the terms “isolation” and “thoughts of cancer” in darker colors including blue, black, and green. The survivor also notes that there are thoughts of cancer and pain, and also includes written-out words of emotions, symptoms, barriers to care and symptom management. This particular survivor chose to use various colors to portray their experience.

The evaluative category was for those drawings that depicted pain as annoying/unbearable. After a careful review of each of the drawings, the research team found no drawing that would be a good fit for the evaluative category.

**Figure 6:**
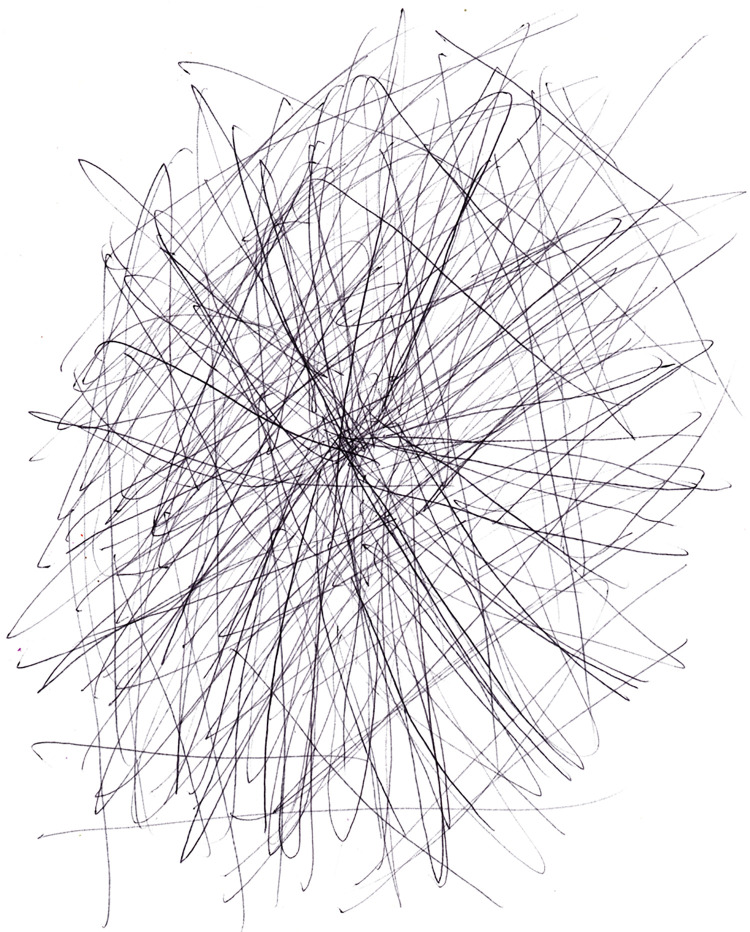
Pain as radiating black lines.

**Figure 7:**
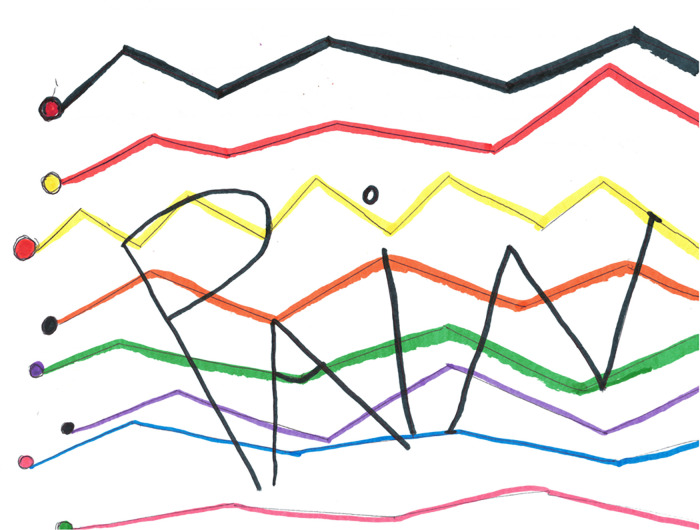
Pain as colored zigzag lines.

Drawings #3 and #11 were placed in the category of “miscellaneous” (see Figures 6, 7, respectively). Drawing #3 shows a dark drawing using a black pen, without any other color, and strong lines radiating out as a web or a maze. It depicts the intensity of pain and fear of reoccurrence. The cancer survivor wrote, “The pain is always there in the middle. It is all black and spreading out. I am always afraid that it will come back and this time it will get me. There is no escape from this blackness.” This particular drawing was shared with the affective category, as it also described the cancer pain in the sensory term of “terrifying.”

Picture #11 (Figure 7) was placed in the miscellaneous category (and shared with the Affective grouping). The descriptors of pain were largely measured by color, which was depicted in the cancer survivor's drawing in a series of colorful zigzag lines to “… give pain a color” … similar to stepping down stairs to “relieve you of pain.” (If) black, (then) meditate until you get it down to white … down to a lot of purple.” This drawing served to depict meditation as a means to reduce pain, to bring the color and thus the level of pain down.

## Discussion

Limited research on the use of standardized assessment tools to evaluate pain intensity among AIs poses difficulty for nurses and other healthcare providers. Although there is no single method for pain measurement, there are some scales that have been shown to be valid measures of pain intensity in general populations and are commonly used: facial scales [FACES® (7)], the Visual Analog Scale (VAS), and the Numeric Rating Scale (NRS). The current study used the FACES scale to measure pain. Facial scales, such as the Wong-Baker FACES® Pain Rating Scale, consist of a spectrum of facial expressions that depict a level of distress and are arranged in hierarchical order (7). Often a numerical value is associated with each facial expression, however the facial scales were developed for children and the expressions are cartoonish. Other pain charts ask patients to identify the location of the pain by visual templates made up of drawings/outlines of the human body (14). Although pain scales such as those described above are commonly used and may be valid among the general population, focused pain assessment and measurement among AIs is limited.

This paper reports on Southwest AI cancer survivors' depiction of physical and non-physical (i.e., psychological, spiritual) pain experiences as illustrated through their drawings. While data on participant attitudes towards existing pain measurement scales was collected, once the pain drawings emerged this study's research interests shifted towards the implications of pain drawings for capturing the more holistic experience of pain that may be missed by mechanistic measurements of pain domains. As a part of a larger study, these focus groups conducted among AI cancer survivors found that experiences with cancer pain were often misunderstood and under-treated, indicating a need for a more comprehensive and culturally-sensitive method for pain assessment for this population. Ethnicity and cultural differences are of practical concern in treatment situations when patients and clinicians are of different ethnic backgrounds. Cultural differences can create boundaries between healthcare providers and patients and frustrate both (15). Under these circumstances, providers may be unable to respond appropriately to the personal needs of their patients and provide reassurance and effective treatment. Inadequate pain treatment may result from the lack of established relationships between patient and clinician, such as acute care settings, where implicit bias may have greater negative influences (3). Problems can occur if there are differences between provider and patient in medical views, beliefs, and social interactions (16). Thus, culture plays an important role in patient communications and provider perceptions of patient pain and other adverse symptoms (17, 18).

Efforts to explore cultural incongruities are reported in a study by Calvillo and Flaskerud (17). In a survey of 60 patients and 60 nurses, differences in Mexican-American and Anglo women's responses to cholecystectomy pain was examined. By comparing the nurses' attribution of pain to the patients' evaluation of the pain, the investigators found that the nurses assigned more pain to the Anglo-American patients than the Mexican-American patients, as well as evaluated their pain as less intense than the Anglo-American patients. The researchers concluded that the nurses' socio-cultural background influenced their perception of patient pain. Other studies have demonstrated effects of incongruent cultural backgrounds on nurses' perception of patient's experiences of pain, pain severity, and their interpretation of pain-relieving strategies (19). Orhan et al.’s systematic review (20) on chronic musculoskeletal pain across all populations, found differences among racial, ethnic or cultural groups with regards to their pain attitudes, perception of illness, self-efficacy, fear avoidance beliefs, and coping strategies for pain. In other studies, sex differences in pain drawings have been observed with women tending to ascribe pain to larger areas of the body (21, 22).

American Indians have a long history of recording their experiences and past histories through storytelling, oral accounts and through visual depiction. Storytelling has been reported as a form of education, entertainment, and as a means to record historical events (9, 23). The study participants' illustration examples presented in this paper harkens back to the era of “ledger art” among AI groups, a style of visual history developed by AI warriors from the Northern and Southern Plains and from the Plateau and Great Basin of the United States (24). Using art to memorialize current events, such as cancer-related pain, is an important means to report and measure pain. This manuscript contributes to the understanding of how AI culture may affect provider-patient communication about pain experiences. In instances where AI cancer survivors express dissatisfaction with existing pain scales, holistic care may offer another option—be it blank paper to allow patients to visualize pain in a way that scales, numbers and words simply cannot do justice. Using holistic approaches such as those reported herein warrant further investigation.

Pain is a multi-dimensional feeling that can be interpreted in a two-dimensional format, as evidenced by the pain artwork created in AI cancer survivors taking part in this study. Their illustrations demonstrate not only pain severity and location, but aspects of the survivor's cultural identity and intergenerational traditions. Color has long been used in AI and Indigenous art for symbolic and spiritual purposes, often with numerous meanings, varying by tribe and region (25–27). For example, for Navajo red may symbolize blood or fire, and yellow can mean fruition (25). Many of these meanings may have been in the minds of cancer survivors and their caregivers when creating their pictures of pain. For the purpose of this study, each participant's color choices were either self-explained in their annotation or inferred by the team of evaluators.

In this study, it is hypothesized that cultural ways of communication and intergenerational influences of artwork led to the depiction of pain in drawings. Each AI cancer survivor's drawing presented is a valid and culturally appropriate depiction of a survivor's pain experience. However, the drawings are based on an individual subjective experience and may not be accurately interpreted without a complimentary detailed description of the drawing that is considered in the context of the individual's situation. Other factors, such as differing cultural backgrounds of the patient and evaluator, must be considered in order to generate valid assessments. Further research is recommended to consider if pain drawings can be incorporated into a new scale or a representative measurement of cancer-related pain experience for individuals. Since the first step in treatment is the assessment of a patient's pain, research must explore the shortfalls of existing scales, particularly among racial/ethnic minorities. Studies targeting other populations used tablets to electronically capture pain drawings on a spatial body template which have shown to be a reliable measurement of acute pain (28). Another study has shown pain drawing on a digital body chart to be a valid measure of chronic neck pain (14), as well as shown promise in clinical assessment and treatment of acute pain (29). However, further investigation into new strategies for reimagining more-open-ended visual tools for pain assessment for the AI community and beyond is needed.

### Implications for holistic clinical practice

This paper, guided by the concept of humanism, reinforces an individualized approach to therapy and care, thus it is important to assess preferred methods of communication among AIs. A humanistic approach understands that an individualized approach to assessment and therapy is needed in order to accurately measure and address pain intensity. For instance, among many AI tribes it is more common to think and express thoughts using traditional storytelling (30), and thus storytelling became an important method used in interpreting the focus group pain drawings, as well as in the larger study. Storytelling is a multidimensional, nonlinear, expression that uses events and relevant factors to help describe or explain emotions or experiences. Because the use of storytelling is the traditional form of communication in this community, it should be the duty of healthcare providers to pay closer attention to this cultural factor as it plays into assessment of pain among patients. American Indian patients' use of storytelling and art provide individual reflection and, in most cases, the former is the preferred method of conveying messages among AIs to others (23). Given this tradition, it would be sensible to assume that AI patients would find it more difficult to measure their pain by simply assigning their pain to one number on a scale, a single phrase of few words, or a facial expression, all of which assume linear thoughts and limited expression.

Better understanding the cancer pain experience adds depth and breadth to the holistic approach of patient care assessment and treatment. A humanistic approach to the assessment of pain in cancer patients can affect pain treatment regimens, and potentially patient satisfaction and outcomes. Enhancing culturally-competent communication with cancer survivors and healthcare providers facilitates and improves holistic clinical practice by utilizing the humanistic approach to care giving.

This study provides evidence that self-expression through color, imagery and written personal accounts offers more accurate depictions of pain for Southwest AI cancer survivors than pain scales alone. This qualitative and visual depiction of pain through art appears to better describe the apparent multidimensional aspects of pain, but would likely still need to be used in tandem with established quantitative pain level assessments. Still, the pain drawing technique could be practically applied in cancer care/healthcare settings in two important ways: (1) as groundwork for the development of an open-response, color-coded pain management index, and (2) as a therapeutic and cultural practice for healing, incorporating personal heritage/history documentation into the long-term cancer symptom management care plan. Pain drawings, such as those seen in this study, may be used to (31): differentiate between new experiences with pain from the old; help distinguish between nociceptive and neuropathic pain to improve treatment outcomes; aid in evaluating a patient's response to a given treatment or therapy; and divide patients into groupings by the area of the body that is painful or bothersome so, where appropriate, specialist support may be sought. Artwork therapies for pain (from cancer, as well as other diseases) (32) are being explored more frequently as routes for not only patient and survivors' expression, but also pain visualizations to help healthcare providers better understand patient pain experience (33) for guiding treatment.

### Limitations of the study

There are some limitations to this study. For example, although the use of art for self-expression and for healing is a commonality shared across many tribes, regions, and cultures, the specific findings of the study may not be generalizable to AIs living outside of the Southwest United States. Reviewer bias is possible due to differing cultural lenses of the non-AI evaluators. Future studies might cross-check reviewer interpretation of pain drawings with participants to confirm intentions, although this is not always possible. Efforts to reduce bias included use of a clear and uniform evaluation structure and initial independent evaluator reviews of the pain drawings and annotations. Another limitation of the study was there was no control drawing. The role of demographics, cancer-type and severity, and comorbid conditions was not evaluated in this study. As such, this study may be seen as a pilot study in the assessment of individualized pain drawings for use in clinical practice.

## Data Availability

The raw data supporting the conclusions of this article will be made available by the authors, without undue reservation.
